# Supplementary material for: Overweight, Obesity, and Depression in Multimorbid Older Adults: Prevalence, Diagnostic Agreement, and Associated Factors in Primary Care—Results from a Multicenter Observational Study
Source: Nutrients. 2025 Apr 21;17(8):1394. doi: 10.3390/nu17081394 (PMC12030450; doi:10.3390/nu17081394)
Supplement: Supplementary file 1 [file nutrients-17-01394-s001.zip › nutrients-3570004-supplementary.pdf]

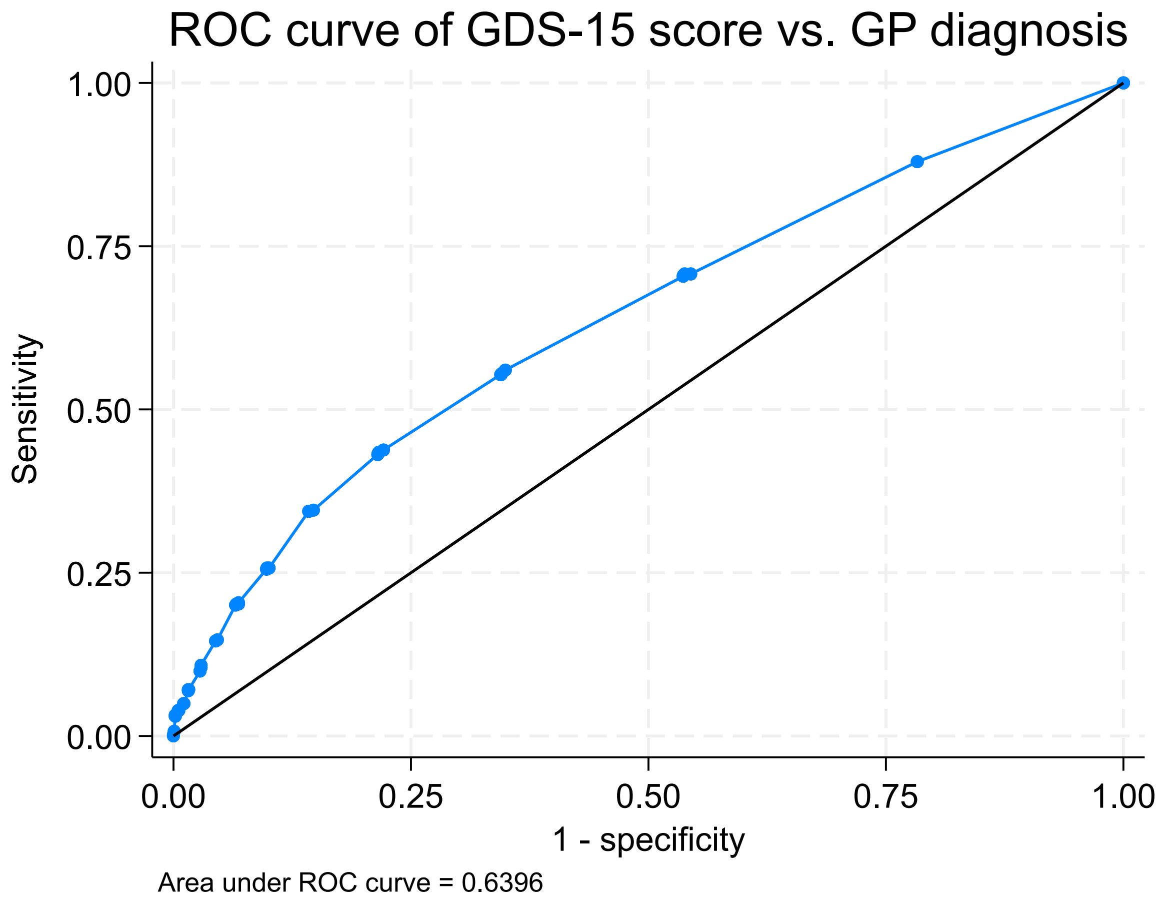

Figure S1. Receiver Operating Characteristic (ROC) analyses

NOTE: Sensitivity = true positive rate; 1-specifity = false postive rate
